# Pre-stroke frailty in patients with first-ever acute stroke: Its associations with stroke subtype and impact on activities of daily living

**DOI:** 10.1016/j.ensci.2026.100626

**Published:** 2026-10-16

**Authors:** Tatsuya Iwasawa, Yoichiro Aoyagi, Yuki Sakamoto, Miho Ohashi, Satoshi Ikeda, Tomonari Saito, Takashi Shimoyama, Satoshi Suda

**Affiliations:** a Department of Rehabilitation Medicine, Nippon Medical School Hospital, Tokyo, Japan; b Graduate School of Medicine, Nippon Medical School, Tokyo, Japan; c Department of Neurology, Nippon Medical School, Tokyo, Japan

**Keywords:** Frailty, First-ever stroke, Activities of daily living, Atrial fibrillation, Cardioembolic stroke

## Abstract

**Objective:**

Few studies have examined frailty in patients with first-ever stroke who were independent in activities of daily living (ADLs) prior to onset. This study aimed to investigate the characteristics of pre-stroke frailty and its impact on ADLs at discharge.

**Methods:**

In this prospective cohort study, 152 patients aged ≥65 years with first-ever stroke admitted to the stroke unit of a university hospital were enrolled. Pre-frailty/frailty was assessed using the Clinical Frailty Scale (CFS). Associations between frailty status and clinical characteristics were evaluated. Multivariate regression analysis identified independent predictors of the Barthel Index (BI) at discharge.

**Results:**

Pre-frailty/frailty was present in 35.5% of patients, and was associated with higher rates of atrial fibrillation (*p* = 0.043) and cardioembolic stroke (*p* = 0.001). Compared with the robust group, the pre-frailty/frailty group had significantly lower BI scores at both admission and discharge, as well as more extended hospital stays. Both groups, however, demonstrated significant improvement in BI at discharge (*p* < 0.001). Multivariate analysis revealed that NIH Stroke Scale score (*p* < 0.001), stroke type (*p* = 0.010), and CFS score (*p* = 0.044) independently predicted BI at discharge.

**Conclusion:**

Pre-stroke frailty is associated with a higher risk of cardioembolic stroke and poorer functional status during hospitalization. Nevertheless, patients with frailty still achieved significant ADL improvement with rehabilitation, underscoring the importance of active rehabilitation strategies in this population.

## Highlight

• Among 152 patients with first-ever acute stroke, 35.5% had pre-frailty or frailty prior to onset.
• The pre-frailty/frailty group showed significantly lower Barthel Index (BI) scores at admission and discharge and had longer hospital stays than the robust group.
• Both groups demonstrated significant BI improvement during hospitalization, indicating that frail patients still benefit substantially from acute-phase rehabilitation.
• Multivariate analysis identified pre-frailty/frailty, stroke severity (NIHSS), and stroke type were independently associated with BI at discharge.
• Pre-frailty/frailty was associated with higher rates of atrial fibrillation and cardioembolic stroke, suggesting that frailty itself may be a risk factor for stroke onset.

## 1 Introduction

As the aging population grows, frailty has gained increasing attention. Frailty is defined as “a state of decreased physiological reserve and increased vulnerability to various stressors; however, it is a clinical condition with reversible characteristics” [1], [2]. Frailty has been identified as a significant factor that increases the risk of death, dependence, and prolonged hospitalization among older individuals. The prevalence of frailty among community-dwelling adults aged 65 and older is approximately 7% according to the Cardiovascular Health Study (CHS) criteria [3] and about 10% according to the Clinical Frailty Scale (CFS) [4], [5].

Frailty has been increasingly investigated in patients with stroke. A meta-analysis of data from patients with acute stroke found that approximately 25% exhibited pre-stroke frailty [6], a higher percentage than in healthy individuals. This suggests that individuals who develop a stroke are more likely to be frail prior to their illness. Additionally, pre-stroke frailty has been independently identified as a factor that negatively impacts quality of life and functional prognosis [6]. Assessing pre-stroke frailty immediately after stroke onset and implementing interventions may improve patient outcomes.

In a previous study, we prospectively examined frailty in individuals with acute stroke in Japan using the CFS [7]. The results indicated that 31% of stroke patients had pre-stroke frailty, a significantly higher prevalence than that observed among older individuals in Japan [3]. We also found that CFS score, NIHSS score at admission, and recurrent stroke were independent predictors of the modified Rankin Scale (mRS) at discharge [7]. However, this study had two major limitations [7]. First, it included patients with recurrent strokes who may have experienced a decline in activities of daily living (ADLs) prior to onset. Since the CFS score varies based on independence in ADLs [8], patients who have previously declined in ADLs are likely to have poorer outcomes. To date, no studies have focused explicitly on first-ever stroke patients who were independent in ADLs before onset. Second, although the modified Rankin Scale (mRS) was used, the Barthel Index (BI) is a more desirable measure of disability.

Evidence regarding the association between frailty and atrial fibrillation remains inconsistent [9], [10]. A recent population-based cohort study reported a higher risk of hospitalization for atrial fibrillation among pre-frail and frail older adults [10]. Given that atrial fibrillation is a major cause of cardioembolic stroke, pre-stroke frailty may also be associated with cardioembolic stroke. However, this association has not been well investigated.

The current study therefore aimed to investigate the characteristics of frailty in patients with first-ever acute stroke and pre-stroke ADL independence, and to assess its association with stroke subtype, BI, and length of stay. We hypothesized that pre-stroke frailty would be associated with a higher risk of cardioembolic stroke, particularly in the presence of atrial fibrillation, and that frailty would predict BI at discharge independent of stroke severity.

## 2 Materials and Methods

### 2.1 Study design and participants

In this prospective cohort study, we recruited consecutive patients with acute stroke admitted to the Stroke Unit (SU) of Nippon Medical School Hospital between June 1, 2022, and July 31, 2023. Patients younger than 65 years, those with recurrent strokes, and those with a premorbid mRS ≥3 were excluded because this study targeted patients whose pre-stroke ADLs were independent before stroke onset.

This study was approved by the Ethics Committee of Nippon Medical School (B-2020-302), and all participants or their family members provided written informed consent before participation. The study conformed to the Strengthening the Reporting of Observational Studies in Epidemiology guidelines.

### 2.2 Clinical characteristics

The registry database included data on age, sex, body mass index, and medical history, including hypertension, dyslipidemia, diabetes mellitus, atrial fibrillation, and ischemic heart disease. Stroke severity was assessed using the National Institutes of Health Stroke Scale (NIHSS) score at admission, on day 7, and at discharge. Data on stroke type (infarct or intracerebral hemorrhage), mRS score at discharge, length of hospitalization, and discharge destination were also recorded. Patients with ischemic stroke were categorized as per the Trial of Org 10,172 in Acute Stroke Treatment (TOAST) criteria [11].

### 2.3 Frailty

Frailty was assessed using the CFS. The CFS is a frailty scale developed by Rockwood et al. and its reliability and validity have been established [8]. For patients admitted to our hospital with stroke, physical therapists obtained information regarding their premorbid condition through interviews. When direct interviews were not feasible due to impaired consciousness or other reasons, relevant information was collected from family members and/or medical records. Patients were scored only when the information was sufficiently clear to allow accurate grading. In this study, CFS scores of 1–3 were defined as robust, 4 as pre-frailty, and ≥5 as frailty.

### 2.4 Activities of daily living (ADLs)

The BI was used to assess ADLs [12]. The BI is scored on a scale of 0–100, with individual items rated on 2- to 4-level scales using increments of 5 points (0, 5, 10, or 15), depending on the activity [13], [14], [15]. Various applications of BI outcomes have been reported, including determining the extent of ADL changes during hospitalization (BI gain) and correcting the ceiling effect of BI gain (BI effectiveness) [16]. BI gain was calculated as *BI at discharge − BI on admission*, and BI effectiveness as *BI gain / (100 − BI on admission)*. In this study, BI at admission and discharge, BI gain, and BI effectiveness were measured and analyzed.

### 2.5 Statistical Analysis

Differences and changes in BI from admission to discharge in the robust and pre-frailty/frailty groups were examined for statistical significance using the Mann–Whitney *U* test. Associations with present illness and stroke subtype were analyzed using the chi-square test. To explore factors related to BI at discharge, the Spearman rank correlation test was performed for continuous variables, and the Mann–Whitney *U* test was used for categorical variables. Subsequently, a multiple linear regression analysis was conducted for variables that were statistically significant in the univariate analysis. We excluded BI at admission from the multiple linear regression analysis to focus on factors influencing the activity level at discharge based on the patient characteristics and physical function level at admission. All analyses were performed using SPSS statistical software (version 27; IBM Corp., Armonk, NY, USA), and a *p*-value <0.05 was considered statistically significant.

## 3 Results

During the study period, 433 consecutive patients with acute stroke were admitted to the SU. Based on the participant selection flow diagram, 152 patients were ultimately included in the final analysis (Fig. 1). The median age was 76 years (interquartile range, 72–84 years), and 59% of the participants were male. The prevalence of pre-frailty (CFS score = 4) was 21.7% (33 patients), and that of frailty (CFS score = 5) was 13.8% (21 patients).

**Fig. 1:**
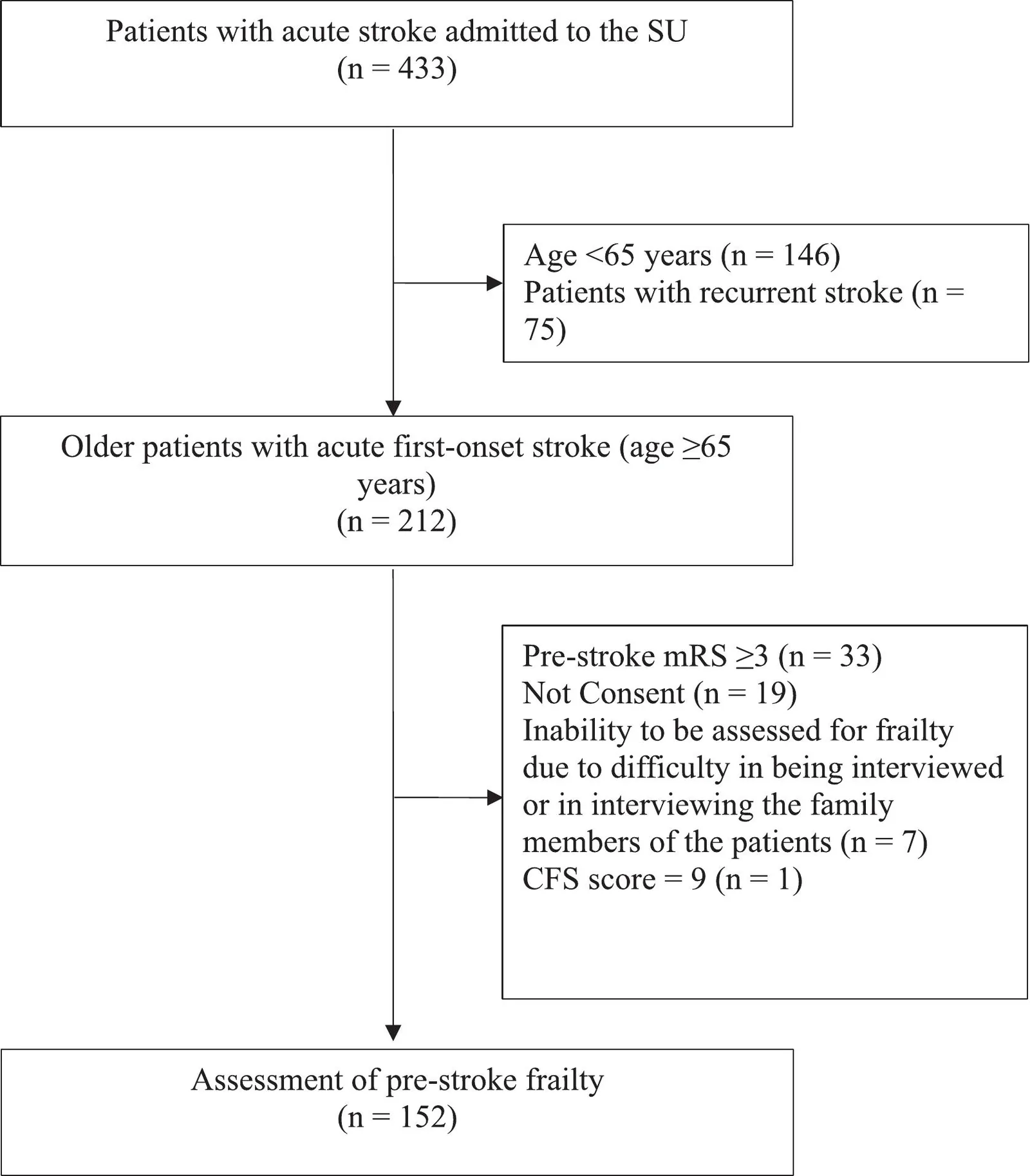
Flow diagram of participant selection CFS, clinical frailty scale; SU, Stroke Unit

Table 1 summarizes the participants' baseline characteristics and the differences according to frailty status. Significant differences were observed in sex, BI (at admission and discharge), NIHSS (at admission and discharge), atrial fibrillation, stroke subtype, mRS score at discharge, and length of hospital stay. The pre-frailty/frailty (CFS score 4–5) group had a higher rate of atrial fibrillation compared to the robust group (22% versus 12%). The risk of cardioembolic stroke was higher in the pre-frailty/frailty group than in the robust group (37% versus 14%).

**Table 1:** Comparison of clinical characteristics of patients in the CFS score 1–3 (robust group) and 4–5 (pre-frailty/frailty group).

Variable	Total	CFS score 1–3 (Robust group)	CFS score 4–5 (Pre-frailty/frailty group)	*p*-value
	*n* = 152	*n* = 98	*n* = 54	
Age, years	76 (72–84)	76 (71–83)	76.5 (74–84)	0.221
Male sex, n (%)	89 (59)	68 (69)	21 (39)	0.001
BMI, kg/m^2^	22.1 (20.1–24.7)	22.2 (20.5–24.4)	21.7 (19.4–24.8)	0.663
Preadmission mRS	0 (0–0)	0 (0–0)	0 (0–1)	0.006
Preadmission CFS score	3 (3–4)	3 (2–3)	4 (4–5)	–
BI on admission	35 (5–60)	40 (12.4–60)	20 (1.25–48.9)	0.016
NIHSS score on admission	4 (2–8)	3 (1–6)	5.5 (2–12.25)	0.005
NIHSS score on day 7	2 (0–5)	2 (0.25–5)	3.5 (2–9)	0.086
Stroke subtype, n (%)				
Large artery atherosclerosis	27 (18)	19 (19)	8 (15)	0.480
Small-vessel occlusion/Lacune	17 (11)	16 (16)	1 (2)	0.007
Cardioembolic	34 (22)	14 (14)	20 (37)	0.001
Stroke due to other determined etiologies	19 (13)	13 (13)	6 (11)	0.701
Stroke due to other undetermined etiologies	36 (23)	24 (24)	12 (22)	0.753
Hemorrhage	19 (13)	12 (12)	7 (13)	0.898
Risk factors, n (%)				
Hypertension	107 (70)	70 (71)	37 (68)	0.874
Dyslipidemia	52 (34)	34 (34)	18 (33)	0.937
Diabetes mellitus	39 (26)	29 (30)	10 (19)	0.190
Atrial fibrillation	24 (16)	12 (12)	12 (22)	0.043
Ischemic heart disease	7 (5)	4 (4)	3 (6)	0.623
Outcome parameters				
mRS score at discharge	2 (1–4)	2 (1–3)	3 (1–4)	0.004
BI at discharge	80 (50–100)	80 (60–100)	65 (20–90)	0.007
BI gain	30 (15–50)	30 (15–50)	30 (10–41.25)	0.239
BI effectiveness	0.6 (0.25–0.93)	0.7 (0.28–1.0)	0.5 (0.1–0.9)	0.069
NIHSS score at discharge	1 (0–4)	1 (0–4)	2 (0–5)	0.194
Days of hospitalization, day	16 (12−21)	15 (10−20)	18 (15–23)	0.003
Discharge from the hospital, home, n (%)	67 (44)	47 (48)	20 (37)	0.161

Data are presented as median (interquartile range) and total number (percentage). BMI, body mass index; CFS, Clinical Frailty Scale; BI, Barthel Index; NIHSS, National Institutes of Health Stroke Scale; mRS, modified Rankin scale.

Supplementary Fig. 1 illustrates the changes in BI from admission to discharge in the robust and pre-frailty/frailty groups. Both groups showed improvement at discharge. BI gain and BI effectiveness did not differ significantly between the groups (Table 1).

Table 2 shows the associations between BI and clinical variables. Age, pre-stroke CFS score, NIHSS at admission, sex, and stroke type were significantly correlated with BI.

**Table 2:** Univariate associations between clinical variables and BI at discharge.

Variable	Category / Unit	BI at discharge (Median [IQR] or ρ)	p-value
Age	years	ρ = −0.196	0.020
BMI	kg/m^2^	ρ = 0.046	0.601
Preadmission CFS score	score	ρ = −0.268	0.001
BI on admission	score	ρ = 0.738	<0.001
NIHSS score on admission	score	ρ = −0.484	<0.001
Sex	Male	80 (60–100)	0.009
	Female	70 (25–90)	
Stroke type	Infarction	80 (55–100)	0.004
	Hemorrhage	47.5 (16.3–80)	
Hypertension	With	75 (46.3–100)	0.548
	Without	87.5 (55–95)	
Dyslipidemia	With	80 (50–100)	0.928
	Without	80 (50–95)	
Diabetes mellitus	With	90 (43.8–100)	0.239
	Without	75 (50–95)	
Atrial fibrillation	With	90 (57.5–100)	0.524
	Without	75 (50–95)	
Ischemic heart disease	With	90 (72.5–100)	0.352
	Without	75 (50–97.5)	

BI, Barthel Index; BMI, body mass index; CFS, clinical Frailty Scale; NIHSS, National Institutes of Health Stroke Scale.

Table 3 presents the results of multiple regression analysis with BI at discharge as the dependent variable. NIHSS (sβ = −0.341, 95% CI: −2.215 to −0.862, *p* < 0.001), stroke type (sβ = −0.199, 95% CI: −33.743 to −4.708, *p* = 0.010), and CFS (sβ = −0.160, 95% CI: −9.593 to −0.144, *p* = 0.044) were independently associated with BI at discharge.

**Table 3:** Results of multiple regression analysis of independent factors related to BI at discharge.

Variable	Sβ	95% CI	p-value
Age	−0.118	−1.204, 0.155	0.122
Sex (female)	−0.085	−15.914, 4.741	0.294
CFS	−0.160	−9.593, −0.144	0.044
Stroke type (hemorrhage)	−0.199	−33.743, −4.708	0.010
NIHSS	−0.341	−2.215, −0.862	<0.001

R^2^ = 0.282. BI, Barthel Index; CFS, Clinical Frailty Scale; NIHSS, National Institutes of Health Stroke Scale.

Additional subgroup analysis was performed to examine the association between frailty status and stroke subtype in patients without a history of atrial fibrillation (Table 4). Among patients without a history of atrial fibrillation, the pre-frailty/frailty group had a significantly higher proportion of cardioembolic stroke compared to the robust group (21% vs 8%, *p* = 0.033). Among the nine pre-frail/frail patients with cardioembolic stroke and no history of atrial fibrillation, atrial fibrillation was newly detected in six patients (atrial fibrillation at admission in two and paroxysmal atrial fibrillation in four). Among the remaining three patients, one had caseous calcification of the mitral annulus and a patent foramen ovale, one had reduced left atrial appendage flow velocity, and one patient had an undetermined cardioembolic source. Among the seven robust patients, atrial fibrillation was newly identified in six patients (atrial fibrillation detected at admission in three and paroxysmal atrial fibrillation in three), while the remaining patient had severely reduced left ventricular systolic function.

**Table 4:** Proportion of stroke subtype by frailty status in patients without a history of atrial fibrillation.

Stroke subtype	Robust (*n* = 86)	Pre-frailty/frailty (*n* = 42)	p-value
Large artery atherosclerosis	18 (21%)	8 (19%)	0.822
Small-vessel occlusion/Lacune	16 (19%)	1 (2%)	0.011
Cardioembolic	7 (8%)	9 (21%)	0.033
Stroke due to other determined etiologies	13 (15%)	6 (14%)	0.901
Stroke due to other undetermined etiologies	19 (22%)	12 (29%)	0.422
Hemorrhage	12 (14%)	6 (14%)	0.960

Among the 128 patients without a history of atrial fibrillation, 86 were classified as robust and 42 as prefrailty/frailty. When the proportions of stroke subtypes were calculated, the prefrail/frail group showed a significantly higher proportion of cardioembolic stroke compared with the robust group (χ^2^ test).

## 4 Discussion

### 4.1 Novelty and key findings

This is the first study to investigate frailty in a prospective cohort of patients with first-ever stroke who were independent in ADLs before onset. Because we recruited individuals with premorbid independence, no patients with CFS scores of 6–8 were identified in our cohort. Our data indicated that pre-frailty/frailty, as assessed using the CFS, was observed in up to 35.5% of individuals. Interestingly, pre-frailty/frailty was significantly associated with atrial fibrillation and cardioembolic stroke, but not with other conventional vascular risk factors such as hypertension, diabetes mellitus, or dyslipidemia. Our findings also confirmed that the pre-frailty/frailty group had higher NIHSS scores, lower BI values at admission and discharge, and longer hospital stays compared with the robust group. The pre-frailty/frailty independently affected ADLs at discharge, even after adjusting for stroke severity and subtype.

### 4.2 Frailty as a potential risk factor for stroke onset

The overall prevalence of pre-stroke pre-frailty/frailty in patients with stroke was 35.5% in our cohort, which was much higher than the 7–10% prevalence of frailty among community-dwelling older adults [3], [4], [5]. Interestingly, pre-frailty/frailty was significantly associated with atrial fibrillation and cardioembolic stroke, but not with a history of atrial fibrillation (Table 1). More interestingly, in non-atrial fibrillation patients, the pre-frailty/frailty group had a higher incidence of cardioembolic stroke compared to the robust group (21% vs 8%, *p* = 0.033) (Table 4). However, atrial fibrillation was newly detected in most patients with cardioembolic stroke who had no prior history of atrial fibrillation. Therefore, this subgroup analysis does not establish an association between frailty and cardioembolic stroke independent of atrial fibrillation. These findings suggest that frailty itself may be a related factor for the development of atrial fibrillation and the cause of cardioembolic stroke. Although our data cannot establish causality, possible mechanisms include impaired left atrial function due to structural cardiac changes [17], a sustained inflammatory state resulting from physical inactivity, hypercoagulability, and endothelial dysfunction [18], [19]. Such mechanisms would distinguish frailty from traditional risk factors and highlight it as a potential contributor to stroke vulnerability. Conversely, atrial fibrillation could worsen the frail state and may represent a marker of frailty. It is also possible that atrial fibrillation and frailty may mutually influence each other, reflecting a bidirectional rather than unidirectional causal relationship (Supplementary Fig. 2). Future studies investigating the biological interplay between frailty, atrial fibrillation, and cerebrovascular events are warranted to validate this hypothesis.

### 4.3 Frailty as a determinant of outcome after stroke

Similar to the present findings, pre-stroke frailty has also been linked to higher mortality and greater stroke severity in stroke populations [20]. However, most included patients with recurrent strokes or pre-existing disabilities, making it difficult to determine whether adverse outcomes reflected frailty itself or prior functional decline. The present study addressed this by focusing solely on first-ever stroke patients who were independent in ADLs before onset, providing clearer evidence that pre-stroke frailty independently determines short-term functional outcomes.

Importantly, even though patients with pre-frailty/frailty demonstrated lower BI scores at both admission and discharge, the degree of improvement during hospitalization—represented by BI gain and BI effectiveness—did not significantly differ from that of robust patients. This suggests that frail patients, despite reduced physiological reserve, retain substantial potential for recovery when provided with appropriate rehabilitation. These findings are consistent with prior evidence from geriatric rehabilitation programs showing that frail inpatients can achieve meaningful functional gains through structured exercise and multidisciplinary care [21].

Our results highlight two key clinical implications. First, frailty should be regarded not merely as a background characteristic but as a modifiable determinant influencing post-stroke recovery trajectories. Early identification of frailty can facilitate individualized rehabilitation planning, targeted nutritional and resistance training interventions, and comprehensive multidisciplinary management. Second, the observed rehabilitation responsiveness among frail patients indicates that frailty should not be used as a criterion to limit rehabilitation intensity. Instead, systematic frailty assessment upon admission could help optimize resource allocation and predict recovery potential more accurately than conventional risk factors alone.

In summary, pre-stroke frailty independently predicts functional outcomes even in previously independent individuals. Recognizing and proactively managing frailty in the acute stroke phase may be crucial to improving recovery and long-term independence in older adults.

### 4.4 Limitations

This study has a few limitations. It was conducted at a single center, which may limit generalizability. Only physical frailty was assessed, without accounting for psychological or social domains. Furthermore, only short-term outcomes were evaluated. Future studies should examine long-term outcomes and test targeted interventions, such as nutritional support and resistance training.

The following is the supplementary data related to this article.

Supplementary Fig. 1 The changes in BI scores at admission and discharge are presented for the robust (CFS score 1–3) and pre-frail/frail (CFS score 4–5) groups. Both the robust and pre-frail/frail groups showed significant improvements in BI scores during hospitalization (*p* < 0.001). Values are expressed as medians with interquartile ranges.

Supplementary Fig. 2 Possible mechanisms that frailty may cause cardioembolic stroke.

## Declaration of generative AI and AI-assisted technologies in the manuscript preparation process

During the preparation of this work, the authors used ChatGPT (OpenAI) to assist with English language editing and improving the clarity of the manuscript. The authors reviewed and edited the output as needed and take full responsibility for the content of the published article.

## CRediT authorship contribution statement

**Tatsuya Iwasawa:** Writing – review & editing, Writing – original draft, Visualization, Validation, Supervision, Resources, Project administration, Methodology, Investigation, Formal analysis, Data curation, Conceptualization. **Yoichiro Aoyagi:** Writing – review & editing, Writing – original draft, Visualization, Validation, Supervision, Methodology, Formal analysis, Conceptualization. **Yuki Sakamoto:** Writing – review & editing, Writing – original draft, Visualization, Validation, Supervision, Resources, Project administration, Investigation, Data curation. **Miho Ohashi:** Writing – review & editing, Writing – original draft, Validation, Project administration, Investigation, Data curation. **Satoshi Ikeda:** Writing – review & editing, Writing – original draft, Validation, Supervision, Formal analysis. **Tomonari Saito:** Writing – review & editing, Writing – original draft, Validation, Resources, Project administration, Methodology, Data curation. **Takashi Shimoyama:** Writing – review & editing, Writing – original draft, Validation, Supervision, Resources, Project administration, Methodology, Formal analysis, Data curation. **Satoshi Suda:** Writing – review & editing, Writing – original draft, Supervision, Resources, Project administration, Methodology, Conceptualization.

## Funding

No funding was received for this study.

## Declaration of competing interest

The authors declare that they have no known competing financial interests or personal relationships that could have appeared to influence the work reported in this paper.

## References

[1] Fried L.P., Tangen C.M., Walston J., Newman A.B., Hirsch C., Gottdiener J. (2001). Frailty in older adults: evidence for a phenotype. J. Gerontol. A Biol. Sci. Med. Sci..

[2] Morley J.E., Vellas B., van Kan G.A., Anker S.D., Bauer J.M., Bernabei R. (2013). Frailty consensus: a call to action. J. Am. Med. Dir. Assoc..

[3] Kojima G., Iliffe S., Taniguchi Y., Shimada H., Rakugi H., Walters K. (2017). Prevalence of frailty in Japan: a systematic review and meta-analysis. J. Epidemiol..

[4] O'Halloran A.M., Hartley P., Moloney D., McGarrigle C., Kenny R.A., Romero-Ortuno R. (2021). Informing patterns of health and social care utilisation in Irish older people according to the Clinical Frailty Scale. HRB Open Res..

[5] Chang C.I., Chan D.C., Kuo K.N., Hsiung C.A., Chen C.Y. (2011). Prevalence and correlates of geriatric frailty in a northern Taiwan community. J. Formos. Med. Assoc..

[6] Burton J.K., Stewart J., Blair M., Oxley S., Wass A., Taylor-Rowan M. (2022). Prevalence and implications of frailty in acute stroke: systematic review & meta-analysis. Age Ageing.

[7] Iwasawa T., Aoyagi Y., Suda S., Ishiyama D., Toi K., Ohashi M. (2023). Prevalence and outcome of pre-onset frailty in patients with acute stroke. Top. Stroke Rehabil..

[8] Rockwood K., Song X., MacKnight C., Bergman H., Hogan D.B., McDowell I. (2005). A global clinical measure of fitness and frailty in elderly people. Cmaj.

[9] Orkaby A.R., Kornej J., Lubitz S.A., McManus D.D., Travison T.G., Sherer J.A. (2021). Association between frailty and atrial fibrillation in older adults- the Framingham heart study offspring cohort. J. Am. Heart Assoc..

[10] Trevisan C., Ceolin C., Vetrano D.L., Petrovic M., Lip G.Y.H., Buchan I. (2025). Frailty increases the risk of hospitalization for atrial fibrillation in older adults: a population-based cohort study. Nutr. Metab. Cardiovasc. Dis..

[11] Adams H.P., Bendixen B.H., Kappelle L.J., Biller J., Love B.B., Gordon D.L. (1993). Classification of subtype of acute ischemic stroke. Definitions for use in a multicenter clinical trial. TOAST. Trial of org 10172 in acute stroke treatment. Stroke.

[12] Mahoney F.I., Barthel D.W. (1965). Functional evaluation: the Barthel index. Md State Med. J..

[13] Granger C.V., Dewis L.S., Peters N.C., Sherwood C.C., Barrett J.E. (1979). Stroke rehabilitation: analysis of repeated Barthel index measures. Arch. Phys. Med. Rehabil..

[14] Gosman-Hedström G., Svensson E. (2000). Parallel reliability of the functional independence measure and the Barthel ADL index. Disabil. Rehabil..

[15] Sonoda S., Tsubahara A., Tajiri H., Igari M., Sawa S., Saito M. (1992). Functional evaluation after Stroke using the FIM (functional independence measure); the relationship between the FIM and the Barthel index, and the contribution of communication scores or social cognition scores. Rehabil. Med..

[16] Koh G.C., Chen C.H., Petrella R., Thind A. (2013). Rehabilitation impact indices and their independent predictors: a systematic review. BMJ Open.

[17] Sun D., Parikh R.R., Wang W., Eaton A., Lutsey P.L., Windham B.G. (2024). Association of left atrial function with frailty: the atherosclerosis risk in communities (ARIC) study. J. Am. Geriatr. Soc..

[18] McKechnie D.G.J., Patel M., Papacosta A.O., Lennon L.T., Ellins E.A., Halcox J.P.J. (2022). Associations between inflammation, coagulation, cardiac strain and injury, and subclinical vascular disease with frailty in older men: a cross-sectional study. BMC Geriatr..

[19] Calila H., Balasescu E., Nedelcu R.I., Ion D.A. (2024). Endothelial dysfunction as a key link between cardiovascular disease and frailty: a systematic review. J. Clin. Med..

[20] Evans N.R., Wall J., To B., Wallis S.J., Romero-Ortuno R., Warburton E.A. (2020). Clinical frailty independently predicts early mortality after ischaemic stroke. Age Ageing.

[21] Coleman S.A., Cunningham C.J., Walsh J.B., Coakley D., Harbison J., Casey M. (2012). Outcomes among older people in a post-acute inpatient rehabilitation unit. Disabil. Rehabil..

