# Supplementary material for: Pre-stroke frailty in patients with first-ever acute stroke: Its associations with stroke subtype and impact on activities of daily living
Source: eNeurologicalSci. 2026 Oct 16;45:100626. doi: 10.1016/j.ensci.2026.100626 (PMC13695303; doi:10.1016/j.ensci.2026.100626)
Supplement: Supplementary file 1 [file mmc1.docx]

**Supplementary Figure 1**

Admission

Admission

Discharge

Discharge

Robust group

Pre-frail/frail group

Barthel Index (BI)

P < 0.001

P < 0.001
